# Personal, Familial and Environmental Determinants of Drug Abuse: A Causal-Comparative Study

**DOI:** 10.5539/gjhs.v7n4p367

**Published:** 2015-01-25

**Authors:** Homeira Sajjadi, Gholamreza Ghaedamini Harouni, Maryam Sharifian Sani

**Affiliations:** 1 Social Determinants of Health Research Centre, University of Social Welfare and Rehabilitation Sciences, Tehran, Iran; 2 Social Welfare Department, University of Social Welfare and Rehabilitation Sciences, Tehran, Iran

**Keywords:** self-esteem, depression, anxiety, responsibility, family environment

## Abstract

**Aims:**

Two purposes were followed in this study: 1) comparing case and control group in eight factors separately and 2) performing a multivariate analysis for identifying risk and protective factors in relation to drug abuse.

**Methods:**

A casual-comparative study was conducted to investigate the study goals. Fifty Cases in a convenient sampling of addicts referring to addiction withdrawal centers and fifty eligible controls (recruited in a randomly sampling) were identified. One-sample independent T-Test for a univariate and Logistic regression model for a multivariate was conducted.

**Results:**

Univariate analysis: addicted group compared with control group, in terms of aggression, easy access to drugs and depression had higher scores and of other factors (self-esteem, religious affiliation, socioeconomic status, family environment and responsibility) cases had lower scores (p<0.05). Multivariate analysis: Easy access to drugs and depression identified as risk factors (OR>1) and high self-esteem, family socioeconomic status and responsibility as protective (OR<1).

**Conclusions:**

Addiction is a multivariate phenomenon and before any intervention, we have to consider personal, familial and environmental factors and separate subjects by them. We can’t give all of addicts the same prescription and follow a drug therapy approach to treat them. Any addict has a unique profile that should be taken into consideration.

## 1. Introduction

Nowadays, drug addiction is one of the serious social problems in many parts of the world which has a high priority for health managers and decision makers. Drug addiction, not only have negative consequences for individuals, but also threat welfare, political stability, economic and social structure of countries (Dostiyan, Bahmani, Azami, & Ali Akbar, 2013). The trafficking (e.g. annual flow of 430-450 tons of heroin into the global heroin market: NUODC, 2014) and abuse of illicit drugs inflict tremendous harm upon individuals, families, and communities throughout the world (Shannon, 2010). Despite a nearly one century efforts to prevent and control of this costly social problem, the number of addicted people is increasing (Rahmdel, 2003). According to the United Nation Organization, there were 220 million of drug addicts in the world by 2005 (Geramian, Akhavan, Gharaat, Tehrani, & Farajzadegan, 2012). National and international reports show that Iran has the highest rates of substance addiction in the world as this population is growing continuously (KeyvanAra et al., 2008; Kheje & Dadgar, 2013; Mohammadi & Shyany, 2008; Mokri, 2002; United Nations Office on Drugs and Crime [UNODC], 2013). Evidence shows that there are about 2 million drug addicts and 6 million recreational addicts in Iran (KeyvanAra, Kiyanpour, & Jianpour, 2008). Based on another report, 1300,000 addicts (9% women and 91% men) have been identified in Iran that mean age of 45% of them is less than 29 and 30% are between 30-39 years old. In this population, Opium with its derivatives and Glass are the most common consumed drugs, respectively (Iran Drug Control Headquarters [IDCH], 2014).

Psychologists view behavior as determined by a multitude of factors including culture, family, social group, lifestyle, environment, behavior skills, thoughts, feelings and physical factors (McMurran, 2005). Obviously, the factors that determine behavior of people can vary from person to person and also may change over time.

To our knowledge, there are many models for understanding and treating addicted persons prior to 1600 AD (e.g. moral, criminal and preternatural); 1600 to 1900 AD (e.g. epidemic and illness); and models appearing since 1900 AD (e.g. personality disorders, learning, Comorbidity, genetic, recovery, biomedical, bio psychosocial and so on) (Miller, 2013). According to this evidence, there is no single explanation of addiction, but it is clear that a number of factors must be taken into account in explaining addiction. These factors lie within three major domains: biological, psychological and social factors (McMurran, 2005).

Based on previous studies, there are some risk and protective factors that can increase risk of drug abuse and addiction. Some of these factors are listed below: low or high self-esteem (Baumeister, Campbell, Krueger, & Vohs, 2003; Jessor, 1987; Wills, 1994; Gerrard, Gibbons, Reis-Bergan, & Russell, 2000; Sharp & Getz, 1996), depression (Mohhamadi, Aghajani, & Zehtabvar, 2011; Van Hasselt, Null, Kempton, & Bukstein, 1993), anomie, feeling exclusion, communicating with addicts (Bagheri, Nabavi, Moltafet, & Naghipour, 2010), family conflict and disruption (Faizallahi, 2008; MehdiPoor Rabori, Nematollahi, & Nouhi, 2012; Parvizi, Ahmadi, & Nasrabadi Nikbakht, 2004), neglect children and drug use in the presence of them (MehdiPoor Rabori et al., 2012), aggressiveness and assertiveness (Hajhasani, Shafiabadi, Pirsaghi, & Bashirpoor, 2011), anxiety and stress (mohhamadi et al., 2011), economic problems, easy access to drugs, smoking, unemployment (Baghiani Moghadam, Fazel Poor, & Rahai, 2008; Parvizi et al., 2004), inability to say “No”, lack of exercise (Baghiani Moghadam et al., 2008), delinquent friends, unsuitable environment (Faizallahi, 2008), freedom from problems and carefree, feel great and powerful, compensation of social limitations, lack of leisure, oppositional tendencies and curiosity (Parvizi et al., 2004). Also, some of the protective factors are family support, religious beliefs (Dew et al., 2008; Farhadinasab, Allahverdipour, Bashirian, & Mahjoub, 2008; Gryczynski & Ward, 2011), Internal and external religion (Templin & Martin, 1999), religious affiliation (Baghiani Moghadam et al., 2008; Benjet et al., 2007; Zargar, Najariyan, & Naame, 2008), socioeconomic status (Bagheri et al., 2010; Benjet et al., 2007), and family solidarity (Latifiniya, Mohheb, & Pishro Kalankesh, 2009). We couldn’t find any study on responsibility and its relation to drug abuse

However, there are a large body of literature about the role of determinant factors of addiction process, any of them considered the risk and protective factors (personal, familial and environmental) simultaneously. There is not just one factor that predisposes an individual to drug use, but a multiplicity of factors can contribute to decision of his/her for drug using. Therefore, it is necessary to do more studies in this context. The first aim of this study was to compare case and control group in eight factors separately. The second aim was to identify adjusted risk and protective factors of drug abuse.

## 2. Method

A casual-comparative study was conducted among 100 participants in Shahrekord city (in the west of Iran) during 2013-2014. Fifty Cases were selected by using convenient sampling among addicts who attended to addiction withdrawal centers and had a history of addiction. Also, simple random sampling was used for selection of fifty eligible controls. The two groups were matched by age and residence.

Data collection tools In order to compare the case and control groups, investigator-constructed questionnaire was used. It was constructed based on eight scales, including religious belongings, F-SES, family environment, easy access to drugs responsibility, Coopersmith self-esteem, Ahvaz aggression, and Beck Depression. This questionnaire has 59 item with eigh domains, including self-esteem (10 item), aggression (10 item), religious belongings (10 item), family socioeconomic status (7 item), family environment (7 item), easy access to drugs (4 item), depression (7 item) and responsibility (6 item). All of the items were answered on a five point Likert scale ranging from strongly agree to strongly disagree. Exploratory factor analysis (EFA) was used to determine construct reliability of the questionnaire. Prior to performing EFA, suitability of data for factor analysis was assessed. The Kaiser-Meyer-Oklin values are presented in Table 1, exceeding the recommended value of 0.6 and the Barlett’s Tests of Sphericity (Pallant, 2010) reached statistical significance, supporting the factor ability of the correlation matrix. Principal components analysis revealed the presence of one component with suggested items in each scale. Explained percent of the variance ofeach component is presented in Table 1. In order of hardship in access to addicted subjects for researchers, data for reliability of structured tools were only gathered through a sample (n= 60) of general population. To verify the accuracy of respondents answering to the questions, correlations between factors were also calculated (Table 2).

**Table 1:** EFA results related to measure latent variables (factors)

*Scales*	*Number of items*	*KMO*	*Barlett’s Tests*		*% explained variance*
			χ^2^	*P*	
Self-esteem	58	0.855	63	0.000	**62.6**
Ahvaz aggression	30	0.815	59.5	0.000	**84.5**
Religious affiliation	10	0.860	68.08	0.000	**89.5**
F-SES	7	0.63	49.4	0.000	**65.03**
Family Environment	7	0.777	45.4	0.000	**70**
Easy access to Drugs	4	0.812	31.09	0.000	**62**
Responsibility	6	0.819	39.04	0.000	**68**
Depression	21	0.897	44.56	0.000	**76**

**Table 2:** Correlation between factors (**p-value<0.001,*p-value<0.03, NS=none significance, r= Pearson’s r)

Factors	r	(1)	(2)	(3)	(4)	(5)	(6)	(7)
Self-esteem (1)	***r***	1						
Aggression (2)	***r***	-.652^**^	1					
Religion (3)	***r***	.228^**^	-.275^**^	1				
F-SES (4)	***r***	.335^**^	-.281^**^	-.031^**NS**^	1			
Family (5)	***r***	.314^**^	-.344^**^	.493^**^	.080^**NS**^	1		
Easy Access (6)	***r***	-.425^**^	.546^**^	-.284^**^	-.192^*^	-.292^**^	1	
Responsibility(7)	***r***	.224^**^	-.275^**^	.568^**^	.095^a^	.587^**^	-.139^**NS**^	1
Depression (8)	***r***	-.597^**^	.623^**^	-.352^**^	-.277^**^	-.430^**^	.445^**^	-.476^**^

### 2.1 Data Analysis

In bivariate analysis, After the assumptions of homogeneity of variances and normality for all variables were met, Chi square for the comparison of proportions and independent-sample T-Test for comparing differences between groups) were used.. The adjusted association of all proposed factors on the youth’s tendency to drug abuse and addiction were examined by multiple logistic regression model. The following independent variables were entered into the model: self-esteem, aggression, religious affiliation, family socioeconomic status, family environment¸ easy access to drugs, responsibility and depression. Level of confidence interval was 95%. Statistical Package for the Social Sciences (SPSS) was used to the data analysis.

## 3. Results

Mean age of the participants in the case group was 27 (SD= 3.16) years with a range of 18 to 33 years and in the control group was 27.4 (SD=3.95) years with a range of 16 to 32 years. The mean of addicted history was 8 (SD=4.09) years with a range of 2 to 5 years As Table 3 shows, there was a significant difference in education levels of two groups. Numbers of subjects from urban and rural areas was nearly equal. In the case group, 36% of the participants did not respond to the drug used question.

**Table 3:** Descriptive statistics

Categorizes	Control group		Addicted group (Case)	
	N	f%	N	f%
Illiterate	2	4.2	13	26
Primary to Diploma	17	35.4	25	50
Diploma and post diploma	14	29.2	10	20
Bachelor or higher	15	31.2	2	4
Chi Square Results	χ(4) = 33.59; *p*=0.001			
Urban	36	75	35	70
Rural	12	25	15	30
Total	48	100	50	100
Chi Square Results	χ(1) = 0.307; *p*=0.58			
Drug used in Case group				
Glass	--	--	1	2
Heroin and Crack	--	--	1	2
Crack and Glass	--	--	1	2
Heroin and Glass	--	--	3	6
Opium Heroin and	--	--	7	14
Opium	--	--	7	14
Heroin	--	--	12	24
Missing	--	--	18	36
Total	--	--	50	100

N: Number of subjects; %f: percent.

In the bivariate analysis, the case and control groups’ status in the eight factors were examined separately. An independent-sample T-Test was conducted to compare the scores of eight factors for the case and control groups. As Table 4 shows, there weresignificant differences between the two groups in the all factors. The magnitude of the differences in the mean of each factor is reported (eta squared).

**Table 4:** Independent T-Tests Results for comparing case and control groups in terms of eight factors

Variable	Group	Mean	Leven’s Test results		T-Test results		*H*_1_	Eta Squared
			*F*	*P*	*T*	*P*		
**Self-esteem**	Case	12.22	0.386	0.536	8.6	0.000	**Accepted**	**0.339**
	Control	17.6						
**Aggression**	Case	19.26	0.051	0.821	7.87	0.000	**Accepted**	**0.301**
	Control	13.9						
**Religious affiliation**	Case	18.62	0.086	0.77	2.7	0.008	**Accepted**	**0.048**
	Control	20.56						
**F-SES**	Case	6.72	0.445	0.502	4.79	0.000	**Accepted**	**0.138**
	Control	8.94						
**Family Environment**	Case	14.18	0.087	0.769	3.02	0.003	**Accepted**	**0.06**
	Control	16.52						
**Access to Drugs**	Case	14.9	0.268	0.606	7.8	0.000	**Accepted**	**0.297**
	Control	10.04						
**Responsibility**	Case	11.34	0.078	0.781	4.37	0.000	**Accepted**	**0.166**
	Control	13.41						
**Depression**	Case	8.56	0.474	0.493	7.63	0.000	**Accepted**	**0.288**
	Control	5.1						

F-SES: Family Socioeconomic status; F: F Ratio; p: p-value; T: T-value.

The multiple logistic regression analysis demonstrated the risk and protective factors of drug abuse and addiction in young adults. The full model which considered all predictors was statistically significant that indicates the model was able to distinguish between addicted and non-addicted participants (). The model as a whole explained the variance in person status between 51.2% (Cox and Snell R square) to 70.8% (Nagelkerke R squared) of, and correctly classified 87% of cases (sensitivity and specificity of the model was 82% and 89.6% respectively). As shown in Table 5, the independent variables which made a unique statistically significant contribution to the model were Self-esteem, family socioeconomic status, easy access to drugs, responsibility and depression.

**Table 5:** Logistic regression predicting likelihood of respondents categorized into case and control group

Predictors	Beta	S.E.	Wald	*P*	Odds ratio	95% CI for Odds Ratio		R*/P**
						Lower	Upper	
**Constant**	-4.25	3.4	1.56	0.211	--	--	--	--
Self-esteem	-0.252	0.084	9.01	0.003	0.777	0.659	0.916	P
Aggression	0.106	0.087	1.49	0.222	1.11	0.938	1.32	R
Religious affiliation	-0.095	0.096	0.972	0.324	0.91	0.754	1.09	P
socio-economic status	-0.341	0.129	6.98	0.008	0.711	0.552	0.916	P
Family Environment	-0.078	0.083	0.893	0.345	0.345	0.786	1.09	P
Access to Drugs	0.307	0.096	10.27	0.001	1.36	1.12	1.64	P
Responsibility	-0.399	0.106	14.12	0.000	0.671	0.545	0.826	P
Depression	0.291	0.125	5.44	0.02	1.34	1.05	1.7	R

* Risk Factor,

** protective factor.

The strongest predictor was easy access to drugs variable, OR=1.36 (95% CI, 1.12-1.64). This indicated that participants in the case group were 1.36 times more likely to report an access to drugs than those who were in the control group, after adjusting for other factors in the model. The odds ratio of 1.34 for depression, indicating that for every additional score of depression, participants in the case group were 1.34 times more likely to have had depression than the control group, adjusting for other factors in the model. Self-esteem (OR=0.777), family socioeconomic status (OR=0.711) and responsibility (OR=0.671) indicates that the control group had more scores than the case group. Remained variables (aggression, religious affiliation and family environment) had no significant effects in the model (see Table 5).

## 4. Discussion

The present study was designed to determine the effect of eight factors on young adults’ tendency to drug abuse and addiction. Firstly, the case and control groups were compared by the eight factors separately and then a multivariate analysis was used to identify the risk and protective factors.

The results of bivariate analysis showed that the case group had a higher scores than the control group in terms of some factors as aggression, easy access to drugs and depression. Also, in terms of other factors, including self-esteem, religious affiliation, socioeconomic status, family environment and responsibility the cases had lower scores. These findings are consistet with most conducted research (Bagheri et al., 2010; Baghiani Moghadam et al., 2008; Baumeister et al., 2003; Benjet et al., 2007; Bry, McKeon, & Pandina, 1982; Dew et al., 2008; Farhadinasab et al., 2008; Gryczynski & Ward, 2011; Jessor, 1987; Newcomb, Maddahian, & Bentler, 1986; Van Hasselt et al., 1993; Wills, 1994; Zargar et al., 2008). Eta squared coefficients showed that the weight of each factor was different as the case and control groups had the greatest and least difference in self-esteem and religious affiliation, respectively (see Table 2). Therfore, a combined model of all factors was designed to identify the risk and protective factors that predispose the persons to become addicted or not. According to the eight factors which entered into the logistic regression model, easy access to drugs and depression identified as the risk factors. These results are consistent with findings of other research (Baghiani Moghadam et al., 2008; mohhamadi et al., 2011; Van Hasselt et al., 1993). High self-esteem, family socioeconomic status and responsibility identified as the protective factors. In contrast to these findings, Gerrard et al., and Sharp & Getzidentified high self-esteem as a risk factor, whereas findings of Bagheri et al. and Benjet et al. confirmed high socioeconomic status as a protective. In the literature, it was not any findings on responsibility.

Also, as the results showed the family environment, religious affiliation and aggression had no significant effect on attitudes to addiction. This finding can be explained as follows: Firstly, an addiction phenomenon among Iranian families is a taboo that they generally have a negative view toward it. Secondly, religious affiliation among the studied target population, especially in Shahrekord city, is high and they are committed to such beliefs. Finally, other variables in the model, including family environment and religious affiliation that had a negative correlation with aggression acted as a buffer and neutralize the effect of aggression.

Studies support the notion that self- aggression is linked with depression. Becker & Lesiak (Becker & Lesiak, 1977) found that severity of depression correlated with covert hostility, including guilt, resentment, irritability and suspicion, but not with overt hostility. Wolfersdorf and Kiefer (1998) showed that inpatients with depression compared with healthy controls had increased levels of inhibited aggression and covert hostility, but did not express the aggression. Goldman and Haaga (Goldman & Haaga, 1995) in a self-report of outpatients with depression found that they hadincreased anger, suppressed anger and fear of expressing anger compared with controls without depression. Brody and colleagues (Brody, Haaga, Kirk, & Solomon, 1999) found more suppressed anger and increased fear of expressing anger in individuals recovered from depression than in healthy controls that could damage their relationships. All of these studies suggested that anger is a prominent feature of depression. As noted above, both expressed and suppressed anger can be a source of conflict and become self-directed.

## 5. Conclusion

Totally, the results of present study showed that personal, familial and environmental factors have to be considered before any intervention and subjects be separated by them. Any addict has a unique profile that should be taken into consideration. All of them can not take the same prescription and follow a drug therapy for treatment.

The findings should be considered in the light of several limitations. Firstly, this study only examined the voluntary addicts who attended to addiction withdrawal centers, not all addicts around Shahrekord city. Secondly, our statistical analyses were based on self-report data not diagnostic tests. Thirdly, because of limited sample size (n=100), any generalization should be done cautiously. It is required that results to repeat in different and broader contexts.
